# Modification of crystal anisotropy and enhancement of magnetic moment of Co-doped SnO_2_ thin films annealed under magnetic field

**DOI:** 10.1186/1556-276X-9-635

**Published:** 2014-11-25

**Authors:** Sagrario M Loya-Mancilla, Pankaj Poddar, Raja Das, Hilda E Esparza Ponce, Ivan L Templeton-Olivares, Oscar O Solis-Canto, Carlos E Ornelas-Gutierrez, Francisco Espinosa-Magaña, Sion F Olive-Méndez

**Affiliations:** 1 Centro de Investigación en Materiales Avanzados, S.C., Cimav, Av. Miguel de Cervantes 120, Complejo Industrial Chihuahua, C.P. 31109 Chihuahua, Chihuahua, Mexico; 2 Physical and Materials Chemistry Division, National Chemical Laboratory, Dr. Homi Bhabha Road, Pune 411008, India

**Keywords:** Crystal anisotropy, Magnetic anisotropy, Thin film, Ferromagnetism, Antiferromagnetism, Magnetic moment, Spin axis, Diluted magnetic oxide

## Abstract

Co-doped SnO_2_ thin films were grown by sputtering technique on SiO_2_/Si(001) substrates at room temperature, and then, thermal treatments with and without an applied magnetic field (H_TT_) were performed in vacuum at 600°C for 20 min. H_TT_ was applied parallel and perpendicular to the substrate surface. Magnetic M(H) measurements reveal the coexistence of a strong antiferromagnetic (AFM) signal and a ferromagnetic (FM) component. The AFM component has a Néel temperature higher than room temperature, the spin axis lies parallel to the substrate surface, and the highest magnetic moment *m* =7 μ_B_/Co at. is obtained when H_TT_ is applied parallel to the substrate surface. Our results show an enhancement of FM moment per Co^+2^ from 0.06 to 0.42 μ_B_/Co at. for the sample on which H_TT_ was applied perpendicular to the surface. The FM order is attributed to the coupling of Co^+2^ ions through electrons trapped at the site of oxygen vacancies, as described by the bound magnetic polaron model. Our results suggest that FM order is aligned along [101] direction of Co-doped SnO_2_ nanocrystals, which is proposed to be the easy magnetization axis.

## Background

Research in semiconducting oxide thin films has fundamental importance due to its wide range of applications in optoelectronics [1], photoluminescence [2], sensing devices [3], etc. Furthermore, experiments with nanomaterials where the electron charge and its spin orientation, regarded as an additional degree of freedom, are together considered to produce new physical effects situated on the field of spintronics, which is a wide research area that offers options to fabricate faster and lower energy consuming solid state devices. In this field, one of the most surprising and potentially claims is that nonmagnetic semiconductors as Ge and GaAs become ferromagnetic by doping with a few percent of 3d transition metals (TM) as Mn [4-6]. One of the most important issues that need to be solved is how to enhance their Curie temperature (*T*_*c*_), which remains far below room temperature (RT). Diluted magnetic oxides (DMO) are wide bandgap oxides as SnO_2_, TiO_2_, ZnO, etc., that can be used as host semiconducting oxides, and doping with most of the 3d transition metals will produce RT ferromagnetism (FM) [7-9]. However, this is not always successful, as nonmagnetic materials can be obtained depending on the synthesis process and crystal characteristics [7-9]. Research on the physical mechanism governing the ferromagnetic order on DMOs has focused mainly on the following aspects: (a) oxygen vacancy (V_O_) defects [10,11], (b) interstitial cations defects [12], and (c) 3d TM doping [13,14]. The concentration of the doping element remains below the percolation limit established by the relation *x*_*p*_ ~2/*Z*_0_, where *Z*_0_ is the coordination number of the cation [15], for SnO_2_*x*_*p*_ ~32 at.% [16], and this concentration is only limited by the solubility limit of the dopant. However, the concentration of dopant cations is chosen, in most of the cases, below 10 at.% to avoid antiferromagnetic (AFM) coupling between neighboring 3d cations. A particular feature observed on DMO thin films is that different saturation magnetizations (M_s_) are obtained depending on the crystallographic direction on which the magnetic M(H) measurement is performed. In other words, M_s_ obtained in different crystallographic directions does not converge at large magnetic fields as conventional ferromagnetic films do [17]. Normally, in DMO out-of-plane M_s_^⟂^ is 2 to 3 times higher than in-plane M_s_^//^[18,19], suggesting that spin-orbit interaction in these materials is very strong and that measured M_s_ is in fact the spin component along a given direction. Furthermore, M(H) loops in DMO are anhysteretic, and maximum values of coercivity reach some teens of oersted (Oe). Research on 3d TM-doped SnO_2_ thin films was highly motivated after Ogale et al. [20], reported a giant magnetic moment of 7.5 μ_B_/Co with a high *T*_*C*_ =650 K on 5 at.% Co-doped SnO_2_ epitaxial thin films. In this paper, we present a detailed study of the evolution of the intensity of the magnetic moment per Co atom (either FM or AFM) on Co-doped SnO_2_ thin films sputtered on SiO_2_/Si(001) substrates at RT followed by thermal treatments in vacuum under magnetic field applied out-of-plane and in-plane configurations.

## Methods

**Figure 1:**
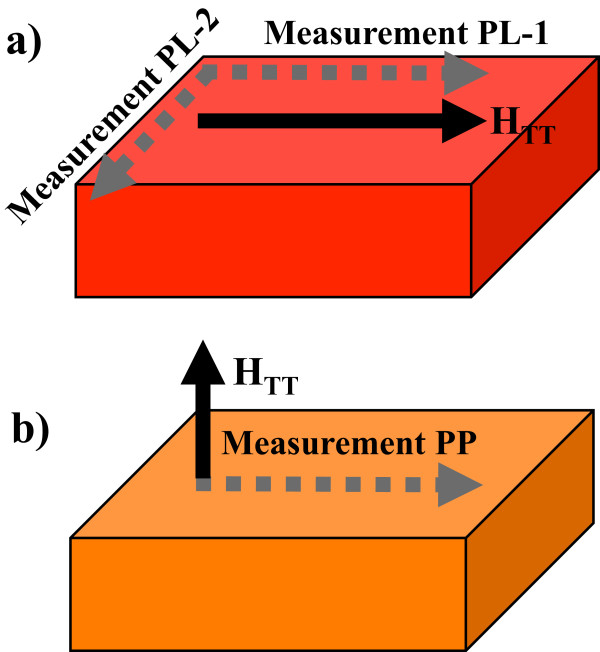
**Configuration of magnetic fields during thermal treatments and magnetic measurements.** Black continuous arrows represent the direction of the applied magnetic field H_TT_ during the thermal treatment, while dashed arrows represent the applied field direction (H_SQUID_) during magnetic measurements. **(a)** Sample PL was measured at two perpendicular directions named PL1 and PL2 and **(b)** sample PP measured only once.

Thin films were elaborated using a sputtering system from Intercovamex (Intercovamex, Cuernavaca, Mor, Mexico), with a base pressure of 1 × 10^−6^ Torr. A home-made SnO_2_ target with Co inclusions was prepared following conventional ceramic methods from high-purity SnO_2_ and Co powder, both with 99% purity from Sigma-Aldrich (Sigma-Aldrich, St. Louis, MO, USA). In a first step, SnO_2_ powders were milled in a high-energy mill 8000D SPEX (Spex Industries, Inc., Metuchen, NJ, USA), for 1 h with ethanol, and then, Co powder was added in stoichiometric proportion to obtain the formula Sn_0.95_Co_0.05_O_2_. The mixture was dried at room temperature and compressed at 4.2 MPa followed by sintering at 1,200°C for 1 h. The obtained pellet had 1-in. diameter. Substrates were 2 × 2 cm^2^ sections cut from an 8-in. diameter SiO_2_/Si(001) wafer. Substrates were first cleaned by an ultrasonic bath for 10 min with ethanol, then rinsed with deionized water, and finally dried with synthetic air. Thin film deposition was achieved using a radio frequency (RF) source at 90 W for 30 min at RT. From a selected sample, four equal 5 × 5 mm^2^ sections were cut in order to apply the thermal treatment (TT) with and without an applied magnetic field (H_TT_). A vacuum tubular furnace [see Additional file 1] was constructed for this purpose and was placed between the poles of an electromagnet. A sample holder inside the furnace allows the placement of the sample parallel (PL) or perpendicular (PP) to the direction of H_TT_. Annealing was performed at 600°C for 20 min, and the intensity of H_TT_ =0.73 T was kept during heating and cooling processes. The samples were named as follows: sample PL, where H_TT_ was applied parallel to the sample surface; sample PP, where H_TT_ was applied perpendicular to the surface; sample NF (no-field) annealed without magnetic field; and as-grown sample is named sample AG. The structural characterization was carried out using grazing incidence X-ray diffractometry (GIXRD), with a fixed incident angle of 0.5° in a PANalytical X'Pert equipment (PANalytical, Lelyweg, Almelo, Holland, The Netherlands). Sample preparation for transmission electron microscopy (TEM) was carried out in a focused ion beam equipment from Jeol (Jeol, Sendai, Japan) and analyzed with a field emission TEM, a Jeol JEM 2200Fs + Cs (Jeol, Musashino, Akishima, Tokyo, Japan). The composition of the film at different points from the surface to the interface with the substrate was obtained by energy dispersive X-ray spectroscopy (EDS) using Oxford spectrometer and INCA software during TEM observations. Magnetic M(H) measurements were performed at 2 and 300 K with a maximum applied magnetic field of 6 T using a superconducting quantum interference device (SQUID) equipment from Quantum Design (SQUID, San Diego, California, USA). For sample PL, two in-plane magnetic measurements were performed: PL1, where the magnetic field from SQUID (H_SQUID_) was applied parallel to the direction of H_TT_; and PL2, where H_SQUID_ is perpendicular to H_TT_. Measurements PL1 and PL2 are perpendicular to each other. Only one in-plane measurement was performed on sample PP, named measurement PP. A diagram depicting these measurements is shown on Figure 1.

## Results and discussion

**Figure 2:**
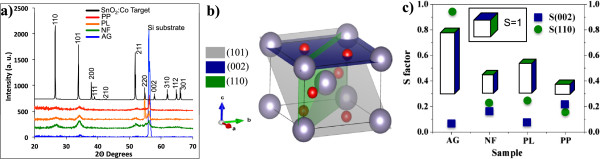
**XRD patterns and grain shape analysis after TT under magnetic field. (a)** XRD patterns for all Co-doped SnO_2_ thin films deposited at RT and with different thermal treatments, the XRD pattern of the target is also shown. **(b)** Schematic unit cell of the SnO_2_ rutile structure showing different planes. **(c)** Variation of the order parameter *S* for all samples; grey blocks represent the suggested grain shape modification, compared to *S* =1 from a pure SnO_2_ crystal.

The GIXRD patterns obtained for all samples including the Co-doped SnO_2_ target are shown on Figure 2a. All the peaks belong to the SnO_2_-rutile structure, and no secondary phases as CoO, Co_3_O_4_, and stannic compounds or pure Co (for the target) were detected. GIXRD from sample AG shows a high substrate signal that covers the intensity of the SnO_2_-rutile peaks; this is due to the random arrangement of the grains in the sample, before any TT. However, perfect randomness is not attained, and there is a trace of crystallinity represented by a small (101) peak, corresponding to the family of planes which is preferred for SnO_2_-rutile thin films (called short-range order) [4], and it may be assumed that no texture is present. Besides, at RT is possible to adduce to the nucleation and crystal growth theory, where a relation between temperature and critical radius r* for stable nuclei is given by:

(1) $$r^{*}=\;\frac{2\sigma \cdot V}{kT\ln S}$$

where *σ* is the surface-free energy, *V* is the theoretical volume of each atom in the crystal, *k* is the Boltzmann constant, *T* is absolute temperature, and *S* is the supersaturation ratio. At RT, there is a larger critical radius that must be reached before crystal growth is achieved. This radius decreases as temperature increases. At this point (higher temperatures), there is a high probability of forming stable nuclei from which grains start to grow. High substrate temperatures make possible the growth of thin films with better crystal quality than those grown at RT. Annealing at 600°C confers enough energy to disordered atoms to promote diffusion allowing crystal rearrangement and crystal growth. In other words, annealing at 600°C induces a crystallization of the SnO_2_-rutile phase. For sample PL, we observed the presence of a peak at 55.11°, corresponding to SnO_2_(110) plane, suggesting an induced texture due to the direction of the magnetic field. This distribution of preferred orientations suggests that an increase in the number of (110) planes is favored by a strong perpendicular component of the spin direction of Co-doped SnO_2_ grains, which may be assumed as the easy magnetization axis. Further structural analysis (not shown) by means of HR-TEM reveals a small increase on the grain size, due to the TT. We found an average grain size of 15 nm for sample AG that increases to 20 nm for sample PL. Analyzing the relative intensities of the peaks of the XRD patterns, the order parameter *S*[21,22] related to the grain shape, which is defined as:

(2) $$S_{\left(hkl\right)C}=\sqrt{\frac{I_{\left(hkl\right)E}/I_{\left(h'k'l'\right)E}}{I_{\left(hkl\right)T}/I_{\left(h'k'l'\right)T}}}$$

where *I*_(*hkl*)*E°*_ and *I*_(*h* ' *k* ' *l* ')*T*_ correspond to experimental and theoretical intensities of a given plane, and *I*_(*hkl*)*E*_ and *I*_(*h* ' *k* ' *l* ')*T*_ correspond to experimental and theoretical intensities for those families of lattice planes which show a particular change. This equation was used to establish the effect of H_TT_ on the morphology of the Co-doped SnO_2_ grains in the film. A pure SnO_2_ crystal has a specific shape in order to minimize the total surface-free energy corresponding to *S* order parameter equal to one, depicted on the inset of Figure 2c. The change on the intensity of certain peaks provides a number to describe morphological changes. Applying Equation 2 to the intensities of (002) and (110) peaks compared to the intensity of (101) peak, which is normally parallel to the substrate surface for SnO_2_ thin films (Figure 2b), it is possible to calculate the order parameter *S* and determine the relative grain shape. This grain shape evolution is attributed to the growth along a component of the spin direction, which is parallel to the direction of H_TT_ during TT. This assumption can explain how grain growth is favored along some defined directions by the direction of H_TT_. In Figure 2c, the relative intensities of the *S* order parameter and a representation of the grain shape for each sample compared to that of pure SnO_2_ grains are shown.

**Figure 3:**
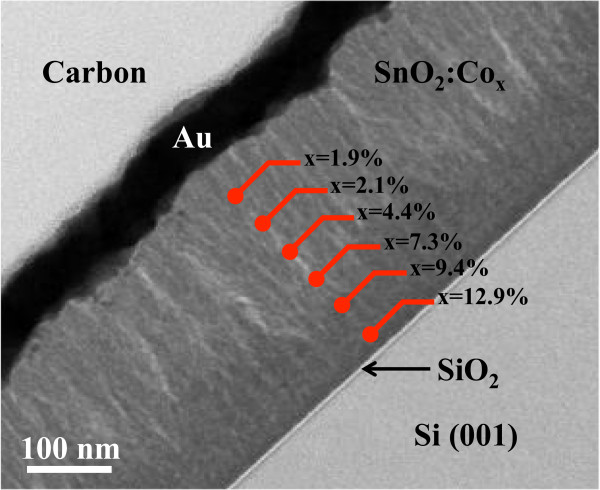
**TEM and EDS analysis for sample PL.** Cross-sectional view of sample PL showing the Co variation on atomic percent (indicated by x in the figure) at different points from the surface to the interface with the substrate. Carbon and Au layers are used as protection barrier and electrical ground for FIB preparation.

Figure 3 shows a TEM cross-sectional view of sample PL where the film thickness is ~300 nm. Punctual EDS analysis, indicated by red points in the figure, was performed during TEM observations in order to evaluate the atomic Co distribution on the film (i.e., *x* on the formula Sn_1−*x*_Co_*x*_O_2_, at.% = *x* × 100). Near to the interface with the substrate, Co concentration is *x* ~13 at.% that decreases as the probe moves towards the surface reaching a minimum value of ~2%. This reduction of Co concentration is attributed to an induced oxidation of the exposed Co particles on the target, during sputtering process, making them harder to erode. The high Co concentration near the interface has a significant effect on the magnetic properties, as it will be further described.

**Figure 4:**
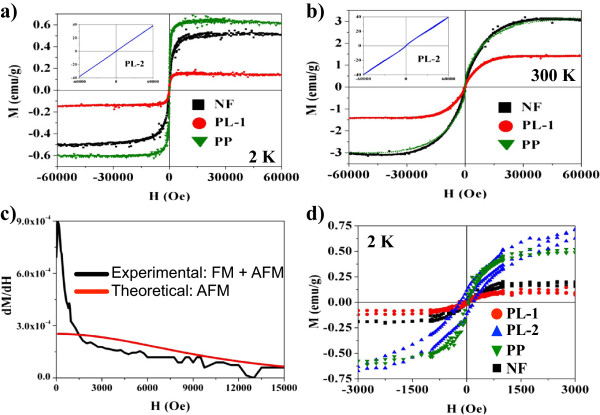
**Magnetization loops obtained at 2 and 300 K.** M(H) loops after subtraction of the diamagnetic component from the substrate; insert: sample PL-2. **(a)** at 2 K and **(b)** at 300 K. **(c)** Derivative of experimental and theoretical M(H) loops for sample PP, evidencing the coexistence of two magnetic components. **(d)** Ferromagnetic component isolated from M(H) hysteresis loops from measurements at 2 K.

**Figure 5:**
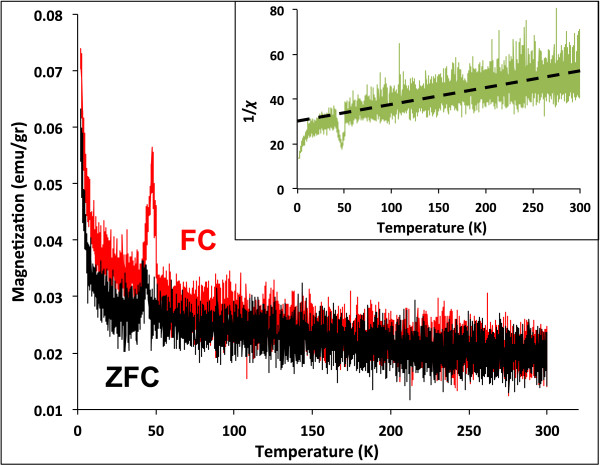
**ZFC-FC performed on sample PP.** Typical ZFC-FC curves observed on DMO system, and the peak at 41 to 47 K is attributed to a minority Co_3_O_4_ AFM nanoclusters. In the inset, an extrapolation of the 1/*X* curve intercepts the temperature axis at a negative value demonstrating that a part of Co atoms is AFM coupled.

To evaluate the effect of H_TT_ on the magnetic properties of the films, magnetic M(H) measurements PL1, PL2, and PP were performed at 2 and 300 K. Figure 4a shows M(H) loops obtained at 2 K, depicting an apparent ferromagnetic and paramagnetic (inset) behavior. A first attempt to elucidate the magnetic behavior of the films was to fit the experimental loops with the Brillouin function, which describes the magnetization process for any FM or AFM material:

(3) $$B\left(J,\;a^{'}\right)=\;\frac{2J+1}{2J}\cot\left(\frac{2J+1}{2J}\right)\cdot a^{'}-\;\frac{1}{2J}\coth\frac{a'}{2J}$$

where *a*^'^ = *µ*_*H*_*H*/*KT*, *µ*_*H*_ is the maximum magnetic moment of Co atoms on the field direction and *J* =1/2 as only spin component appears. Quenching effect on 3d TM makes *L* =0; *J* = *L* + *S*. However, a closer look at the origin of the M-H plane, leads to define two magnetic phases as an abrupt change on the slope has detected. To make evident in this fact, the derivative of the first quadrant, of both, the experimental and the fitted Brillouin curves, was obtained for all measurements. In Figure 4c, the results for sample PP are shown. The derivative of the experimental measurement decreases abruptly from 0 to 1,500 Oe, then it decreases softly with almost the same rate than that of the fitted curve, and this is the demonstration of the coexistence of two magnetic phases. To elucidate the nature of the two components, we focus on measurement PL2, which seems to be paramagnetic; however, paramagnetic susceptibility varies with temperature through the expression, *X* = *C*/*T*, where *C* is the Curie constant. PL2 measurements at 2 and 300 K have the same slope, and then, paramagnetic behavior is discarded. Furthermore, the solubility limit of Co on SnO_2_ is 2 at.% [18] thus, near to the interface with the substrate where Co concentration is ~13 at.%, an AFM coupling is expected. Perpendicular AFM susceptibility (*X*_⟂_) is not temperature dependent below Néel temperature (*T*_N_). AFM coupling of Co atoms in the rutile structure may be similar to superexchange interaction on CoO where *T*_N_ =292 K [23]. Equation 3 was used to evaluate the AFM magnetic moment (*m*) per Co atom, by fitting to the experimental measurements NF, PL1, and PP. The maximum AFM moment *m* = 7.01 μ_B_/Co was obtained on sample PL. For measurement PL2, we used the equation for perpendicular anisotropy in an antiferromagnetic material:

(4) $$X_{⊥}=\frac{C}{2\theta_{P}}$$

where *θ*_*P*_ is the paramagnetic Curie temperature (calculated from 1/*X* vs. *T* plot, insert Figure 5) and *C* the Curie constant *C* = *Nμ*^2^/3*Ak*, where *N* is Avogadro number, *μ* is the magnetic moment per atom, and *A* is the atomic weight. The slope of the AFM component is equal to *X*_⟂_ from where the AFM moment *m* =6.54 μ_B_/Co at. is obtained, which is similar to the one obtained on PL1 measurement (7.01 μ_B_/Co). From this analysis, the direction of PL2 measurement corresponds to an induced spin axis of the AFM pairs, due to the effect of TT under H_TT_. On sample PP, the AFM moment decreases to 4.96 μ_B_/Co at. suggesting that AFM coupling is more stable when spin axis is oriented parallel to the substrate surface. It may be observed how AFM component almost vanishes at 300 K, which is a temperature barely lower than *T*_N_ in our samples. Using the intensity of the magnetic moments of each sample and the *m* per Co atom, we found that 46% of the total Co atoms are AFM coupled.

In a second step, to quantify the FM component per Co atom, the Brillouin fit was subtracted from the experimental measurements to obtain a FM loop as shown on Figure 4d. The total magnetic moment of the sample is divided by the remaining amount of FM Co atoms to obtain the individual *m*. In Table 1, the *m* for all samples at 2 and 300 K is shown. The source of the FM component is attributed to the BMP model on DMO [24]. Magnetic moments from measurements PP and PL2 at 2 K are *m* =0.31 and 0.42 μ_B_/Co at., which decreases to 0.018 and 0.12 at 300 K, respectively. The *m* of sample PP remains more stable against temperature perturbations demonstrating that the FM *m* has a strong component perpendicular to the substrate.

**Table 1:** **Magnetic moments** ***m*** **in μ**_**B**_**per Co at. obtained from all measurements at 2 and 300 K**

**Measurement**		**NF**	**PL-1**	**PL-2**	**PP**
AFM moment	2 K	5.39	7.0119	6.5466	4.96
FM moment	2 K	0.0629	0.0642	0.3125	0.4285
	300 K	0.0320	0.0178	0.0180	0.1216

In Figure 5, the ZFC-FC measurement performed on sample PP is shown and the increasing magnetization with decreasing temperature has been observed in other DMO systems [25,26], similar to that of a superparamagnetic system with the difference that there is no any observed blocking temperature. The peak observed at 41 to 47 K can be attributed to a minority of Co_3_O_4_ precipitates undetectable by XRD experiments. The 1/*X* graph presented on the insert shows that the extrapolation of the straight dotted line intercepts the temperature axis at a negative value of *T*, corresponding to the paramagnetic Curie temperature *θ*_*P*_.

**Figure 6:**
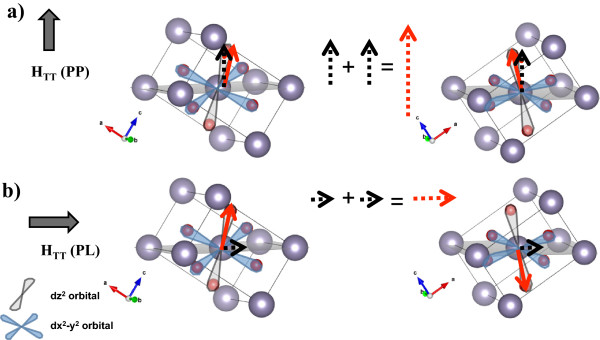
**Proposed model indicating the location and orientation of the spin on the orbital dz**^**2**^**.** Red continuous arrows represent the location and orientation of the spin, and black dashed arrows represent the spin components considered in the obtained FM moments. Red dashed arrows give a qualitative indication of the magnitude of the ferromagnetic moment obtained for each studied case. **(a)** For sample PP, as the spin-orbit interaction is very strong, the vertical component of the spin will align with the applied magnetic field during the TT. **(b)** For sample PL, the horizontal component will align with the direction of the field during TT and the total component is smaller than that of **(a)**.

Crystal field theory (CFT) was used to explain these results in agreement with the anisotropy feature in DMO thin films. For schematic purposes, in Figure 6, a Co^2+^ ion is placed at the center of the octahedron formed by O atoms on the rutile cell. The 3d orbitals of the Co atom according with the CFT are dz^2^ and dx^2^-y^2^, which are degenerated, in a higher energy level than orbitals d_xy_, d_xz_, and d_zx_. Mimaki et al. [27] studied the bond character of rutile type on SiO_2_, GeO_2_, and SnO_2_, and they observed that the ratio of the electron density of M-O equatorial distances/M-O axial distances (where M is Si, Ge, or Sn) decreases when increases the atomic number of the cation. Then, for SnO_2_, the electron density is higher on the dz^2^ orbital and only one unpaired electron will be occupying in this orbital. The FM moments per Co at. are very small, suggesting that the Co atoms have a low spin configuration. This fact is in agreement with the measurements that a higher magnetic moment was obtained for sample PP, this is because dz^2^ orbital axis forms an angle of 34.1° with SnO_2_(101) plane (which remains parallel to the surface), and we assume a very strong spin - orbit coupling. With the TT applied in the PP configuration, the vertical component of the spin is aligned in the same direction than that of the magnetic field. The contribution of the possible random orientations of the SnO_2_ cells is represented in Figure 6a. For PL configuration, the alignment of the spins is in a manner that the horizontal components are parallel to the applied field of the TT, as shown in Figure 6b, and the addition of all these components leads to a total magnetic moment lower than that of the PP configuration. These results can be compared with monocrystalline thin films (e.g., epitaxial films on r-cut sapphire substrate), where the spin components can be better appreciated, and different magnetizations are obtained for PL and PP configurations.

## Conclusions

Polycrystalline Co-doped SnO_2_ thin films were grown by RF sputtering at room temperature. Crystallinity of the films was improved by thermal treatment at 600°C with and without an external magnetic field. The relative intensities of (002) and (110) peaks of the XRD patterns were compared with the intensity of the (101) peak, through the order parameter *S* related to the shape of the nanocrystals. The thermal treatment under magnetic field changed the shape of the crystals, as growth is favored along the direction where the spin is aligned. The analysis of magnetic properties resulted in the observation of two magnetic phases: ferromagnetic (FM) and antiferromagnetic (AFM), where AFM component has a Néel temperature barely higher than RT arising from the coupling between Co atoms in the region near to the interface with the substrate, where Co concentration is ≈ 13 at.%, which is higher than the solubility limit of Co on SnO_2_. Moreover, a modification of crystal anisotropy due to the thermal treatment under magnetic field was observed, enhancing the FM moment for films where the magnetic field during thermal treatment was applied in a direction perpendicular to the substrate surface. The FM moment produced by Co ions arises from the interaction of these ions through the spherical orbit of the electron on the polaron produced by the oxygen vacancy. As the intensity of the magnetization depends on the direction in which the measurement is done and as dz^2^ orbital has the highest probability to contain the unpaired electron, then, we suggest that the spin is perpendicular to the axis of this orbital and parallel to the [101] direction. Proposing that this direction [101]_SnO2_ is the easy axis of magnetization and that magnetization measured along any direction corresponds to the spin component.

## Competing interests

The authors declare that they have no competing interests.

## Authors' contributions

SMLM participated in the thin films growth, data collection, and interpretation. She also worked on the manuscript. PP and RD gave the facilities and performed the magnetic measurements. HEEP participated in the thin films growth and gave equipment facilities. ILTO constructed the vacuum tubular furnace for thermal treatments. OOSC participated in the thin films preparation for TEM analysis and equipment facilities. CEOG performed TEM observation and EDS analysis. FEM participated on the manuscript and SFOM participated in the thin films growth, the data interpretation, and the work on the manuscript. All authors read and approved the final manuscript.

## Authors' information

SMLM is a Ph.D. student in materials sciences at CIMAV. PP is a senior researcher at the National Chemical Laboratory (NCL) in India, and RD is a Ph.D. student at NCL. The rest of the authors work at Cimav Chihuahua. ILTO works as a technical engineer for general laboratory support. OOSC is in charged for the TEM sample preparations using FIB. CEOG performs the TEM observations. FEM is a professor researcher working with the theoretical calculations, and HEEP is a researcher working on the thin films by sputtering. SFOM is a experimental researcher and head of the group.

## Supplementary Material

Additional file 1 **Tubular furnace.** Schema showing the tubular furnace used for the TT under magnetic field (H_TT_). The sample is placed at the interior of the tubular ceramic element parallel or perpendicular to the direction of H_TT_. Click here for file
