# Supplementary figures and images for: Modification of crystal anisotropy and enhancement of magnetic moment of Co-doped SnO2 thin films annealed under magnetic field
Source: Nanoscale Res Lett. 2014 Nov 25;9(1):635. doi: 10.1186/1556-276X-9-635 (PMC4256971; doi:10.1186/1556-276X-9-635)

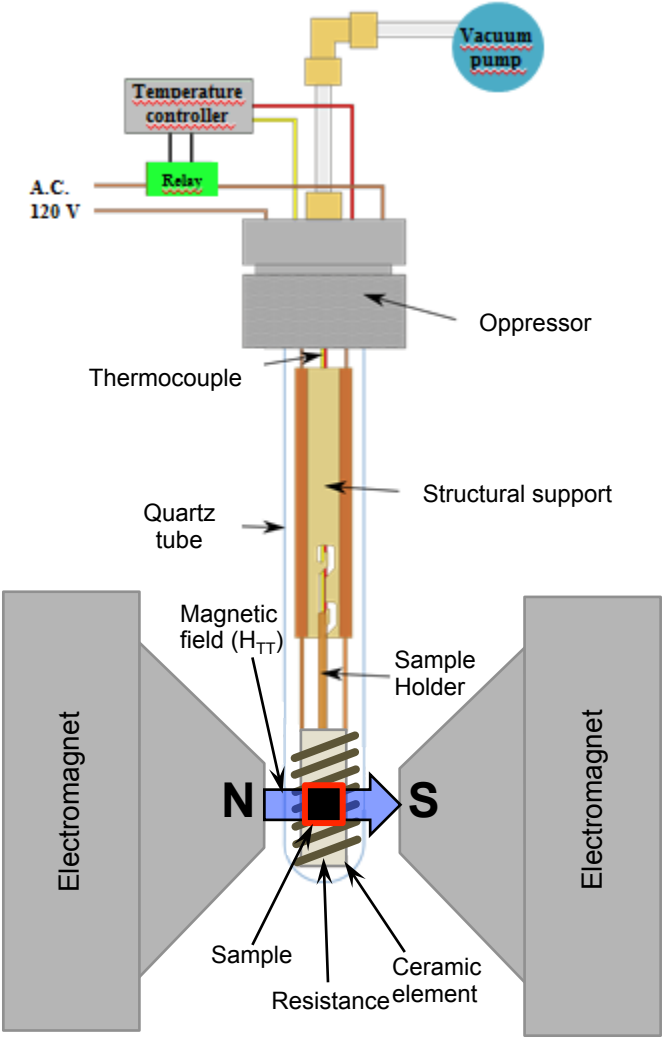

Supplement: Additional file 1 — Tubular furnace. Schema showing the tubular furnace used for the TT under magnetic field (HTT). The sample is placed at the interior of the tubular ceramic element parallel or perpendicular to the direction of HTT. [file 1556-276X-9-635-S1.pdf]
